# Resting-State fMRI Activity Predicts Unsupervised Learning and Memory in an Immersive Virtual Reality Environment

**DOI:** 10.1371/journal.pone.0109622

**Published:** 2014-10-06

**Authors:** Chi Wah Wong, Valur Olafsson, Markus Plank, Joseph Snider, Eric Halgren, Howard Poizner, Thomas T. Liu

**Affiliations:** 1 Center for Functional Magnetic Resonance Imaging, University of California San Diego, La Jolla, CA, United States of America; 2 Department of Radiology, University of California San Diego, La Jolla, CA, United States of America; 3 Department of Bioengineering, University of California San Diego, La Jolla, CA, United States of America; 4 Institute for Neural Computation, University of California San Diego, La Jolla, CA, United States of America; 5 Departments of Neuroscience and Psychiatry, University of California San Diego, La Jolla, CA, United States of America; 6 Graduate Program in Neurosciences, University of California San Diego, La Jolla, CA, United States of America; 7 Neuroscience Imaging Center, University of Pittsburgh, Pittsburgh, PA, United States of America; Wake Forest School of Medicine, United States of America

## Abstract

In the real world, learning often proceeds in an unsupervised manner without explicit instructions or feedback. In this study, we employed an experimental paradigm in which subjects explored an immersive virtual reality environment on each of two days. On day 1, subjects implicitly learned the location of 39 objects in an unsupervised fashion. On day 2, the locations of some of the objects were changed, and object location recall performance was assessed and found to vary across subjects. As prior work had shown that functional magnetic resonance imaging (fMRI) measures of resting-state brain activity can predict various measures of brain performance across individuals, we examined whether resting-state fMRI measures could be used to predict object location recall performance. We found a significant correlation between performance and the variability of the resting-state fMRI signal in the basal ganglia, hippocampus, amygdala, thalamus, insula, and regions in the frontal and temporal lobes, regions important for spatial exploration, learning, memory, and decision making. In addition, performance was significantly correlated with resting-state fMRI connectivity between the left caudate and the right fusiform gyrus, lateral occipital complex, and superior temporal gyrus. Given the basal ganglia's role in exploration, these findings suggest that tighter integration of the brain systems responsible for exploration and visuospatial processing may be critical for learning in a complex environment.

## Introduction

Across a range of learning and memory tasks, the level of performance has been found to vary greatly across individuals [1]–[5]. Prior studies have shown that various measures of brain anatomy and physiology can be used to predict individual variations in performance. For instance, Erickson et al. [2] have shown that individual variations in striatal volume strongly correlate with individual differences in learning a complex video game. Similarly, Vo et al. [1] found that the spatial pattern of T2* weighted magnetic resonance (MR) images in the dorsal striatum at the initial stage of learning can be used to predict subsequent learning performance in a video game.

A growing number of studies are finding that resting-state functional magnetic resonance imaging (fMRI) measures of brain activity, which are based on intrinsic fluctuations in the blood oxygenation level dependent (BOLD) signal, can also be used to predict performance across individuals [1], [3], [6]–[12]. In resting-state fMRI, the correlation between BOLD signals between different brain regions serves as a measure of functional brain connectivity [11]. Functional networks can then be identified by examining the spatial pattern of connectivity. For example, Seeley et al. demonstrated that functional connectivity in the lateral parietal areas of the executive control network was correlated with executive task performance measured outside the scanner [12]. Hampson et al. [9] found that connectivity between the default mode network (DMN) and the task positive network (TPN) can be used to predict working memory performance, while Cole et al. [7] found that whole brain connectivity with the lateral prefrontal cortex can predict fluid intelligence.

In addition to functional connectivity, an increasing number of studies are finding that measures of the variability of the BOLD signal can reflect differences in cognitive performance, as well as changes in brain state associated with disease and aging [4], [8], [10], [11], [13]–[21]. For instance, Zou et al. [10] reported that the amplitude of low-frequency fluctuations (ALFF) of the resting-state BOLD signal can predict working memory performance. Yang et al. [17] have shown that the variability of the resting-state global brain signal is greater in patients with schizophrenia as compared to matched controls. In a study comparing younger and older adults, Garrett et al. [8], [13], [14], [20], [21] found an age-related decrease in BOLD signal variability (defined as the standard deviation of the BOLD signal). Furthermore, an increase in BOLD signal variability was found to be associated with better performance in cognitive tasks. From their studies, Garrett et al. concluded that BOLD signal variability can represent aspects of dynamic brain function that are not reflected in task-related mean BOLD signal changes, with the level of variability potentially serving as a reflection of the robustness, efficiency, and adaptability of underlying neural networks. In addition, variability may be linked to the level of dopamine, which is thought to be a key agent in determining the dynamic capacity of neuronal systems [20]. Overall, the various findings suggest that BOLD signal variability can serve as an important indicator of brain function, with further work needed to better understand the mechanisms that give rise to differences in variability.

In this work, we build upon the prior findings to determine whether resting-state fMRI measures (BOLD signal variability and functional connectivity) can be used to predict performance in an experimental paradigm that involves unsupervised learning in a large-scale immersive virtual reality (VR) environment. Unsupervised learning refers to learning that is self-supervised without explicit teaching [22], and is the type of learning that is often employed in real-world environments. The use an immersive VR environment (in which subjects can move around) enables the study of unsupervised learning in an experimental setting that allows for the interactions and movement that occur in a real-world environment. In a recent study, Snider et al. [23] tested the ability to recall object locations on the second day of an immersive VR experiment in which unsupervised learning of the environment and object locations occurred on the first day. They found that object location recall success varied across individuals and that the degree of success was predicted by the strength of spatial maps formed during the unsupervised learning phase. For this study, we hypothesized that resting-state fMRI measures in brain regions associated with learning and memory (such as the basal ganglia and hippocampus) would also be predictive of individual performance and tested this hypothesis using subjects from the study [23].

## Methods

### Experimental protocol

**Figure 1:**
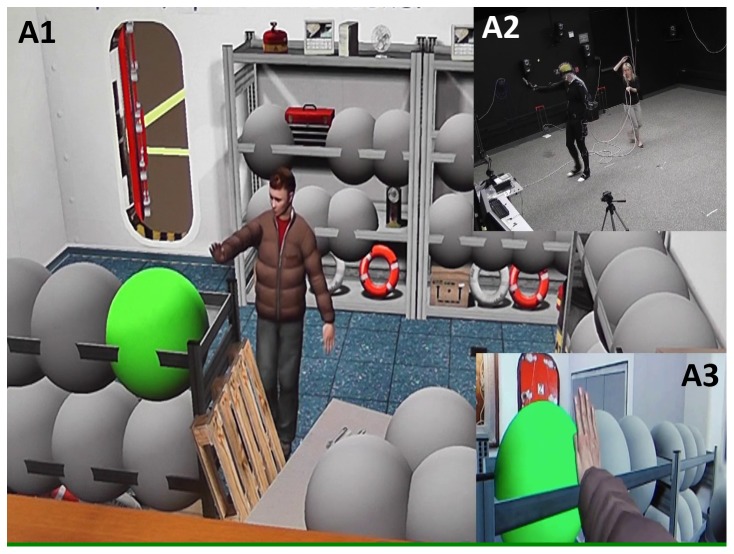
Full immersion VR experiment. The virtual environment (A1, bird's-eye view) is rendered in real time (A3, ego view) and shown to the subject via a high resolution head-mounted display (A2, physical environment).

The University of California San Diego Institutional Review Board approved this study, and thirteen right-handed healthy volunteers participated in the virtual reality portion of this study after signing informed consent documents (3 females, age [mean ± std]  = 25±4 years). The participants did not suffer from acute physical illness, substance abuse or dependence, did not exhibit a history head injury leading to a loss of consciousness, and did not have a history of major psychiatric or neurological illness. Participants abstained from the usage of caffeine, nicotine, and alcohol prior to the scan sessions. Each subject participated in large-scale immersive virtual reality environment exploration (Fig. 1) over two consecutive days (one visit per day, each visit lasting around two hours). In each visit, subjects wore a panoramic high resolution head-mounted display (Sensics xSight 6123, Sensics Inc.) and walked around a virtual reality environment that was a richly textured room (approximately 4 m×5 m, same size as the real world space that the subjects walked in) containing 39 objects placed on shelves, tables, and the floor [24]. Movements of the limb, torso, and head were tracked with a 24-camera 3D tracking system (PhaseSpace Inc.). The 24 cameras were positioned on the ceiling, walls and floor of a 7.5 m×7.5 m×2.9 m room for even coverage and accurate motion tracking over the 4 m×5 m space used in the experiment.

The first visit was dedicated to exploration, and the second visit was used to test the subject's memory of the environment. The subject's naiveté about the memory aspect of the experiment was maintained during the first visit such that recall of the environment during the second visit relied on unsupervised learning. During the first visit, each subject was asked to freely explore the virtual room for 10 minutes. After this initial free exploration was completed, five blocks of tasks were performed. In each block, the 39 objects were covered with an opaque bubble. At a pre-specified time, one of the bubbles would turn green. Subjects were instructed to walk over to the green bubble and touch it. The bubble would then disappear and reveal the object underneath. As a cover task, the subject was told to briefly observe the object and rate how interesting they felt the object was using a virtual sliding scale that appeared in front of them. After each block, the subject would have walked to and rated all 39 objects. For each of these five blocks, the order of bubbles turning green was randomly varied, but each object remained in the same location.

During the second visit, each subject participated in five blocks of tasks with each block lasting for 5 - 8 minutes. The virtual reality environment was identical to the one in the first visit. Before each block, one third of the objects (chosen at random) were shuffled to a new location. Upon revealing an object (by touching the green opaque bubble), the subject was asked to determine whether or not the object had been in that location during the first visit. For each subject, the performance score of the unsupervised learning task was defined as the percentage of correct judgments across all blocks in the second visit. The performance scores of the individual subjects are listed in Table 1.

**Table 1:** Performance scores and head motion of the individual subjects.

Subject index	Performance score (%)	Average Frame Displacement (mm)
1	95.9	0.081
2	85.64	0.058
3	78.75	0.062
4	76.6	0.102
5	90.06	0.105
6	83.59	0.085
7	80.13	0.100
8	88.46	0.108
9	92.27	0.088
10	90.48	0.097

Ten out of the thirteen subjects were able to return and participate in an MRI scan session six to twelve months after the virtual reality visits (two subjects moved out of town and one subject did not respond to our follow up contacts). Each scan session consisted of: (1) a high-resolution anatomical scan, (2) two 8 minute eyes-open resting-state scans and (3) a field map to measure magnetic field inhomogeneities. For the resting-state scans, subjects were instructed to lie still in the scanner and to maintain attention on a yellow fixation cross located at the center of a blue background.

### MR data acquisition

Imaging data were acquired on a 3 Tesla GE Discovery MR750 whole body system using a 32 channel receiver coil (Nova Medical). High resolution anatomical data were collected using a magnetization prepared 3D fast spoiled gradient (FSPGR) sequence (TI = 600 ms, TE = 3.1 ms, flip angle  = 8 degrees, slice thickness  = 1 mm, FOV  = 25.6 cm, matrix size  = 256×256×176).

Whole brain BOLD resting-state data were acquired using multiecho simultaneous multislice (MESMS) echoplanar imaging (EPI). The acquisition used a 2.5-fold phase encode acceleration factor and a blipped-CAIPI EPI k-space trajectory [25]. Three sagittal slices and three echoes were collected per RF excitation to achieve 2 mm^3^ isotropic resolution with whole brain coverage (FOV = 20 cm, 100×100 matrix, 72 slices). Other acquisition parameters were: TR = 2 s, TEs = 15.5 ms, 36.7 ms, 57.9 ms and FA = 30°. During each eight minute resting-state scan, 240 functional volumes were acquired. To reconstruct the images, we used SENSE reconstruction with a fast Conjugate Gradient Toeplitz-based iterative algorithm [26]. It was regularized with an in-plane spatial roughness penalty to achieve an effective FWHM of 1.25 voxels. In this paper, only the second echo BOLD data (36.7 ms) were considered.

A field map was acquired using a gradient recalled acquisition in steady state (GRASS) sequence (TE1  = 6.9 ms, TE2  = 8.9 ms), with the same in-plane parameters and slice locations as the BOLD resting-state data. The phase difference between the two echoes was then used to estimate a field map for magnetic field inhomogeneity correction. The field map was used to warp the coil sensitivities, used in the SENSE reconstruction, to the same spatial coordinate system as the MESMS BOLD data. This was needed due to the phase encode acceleration difference between the coil sensitivity and BOLD data acquisitions.

### MR data processing

AFNI and FSL were used for MRI data pre-processing [27]–[29]. The high resolution anatomical data were skull stripped and segmentation was applied to estimate white matter (WM), gray matter (GM) and cerebral spinal fluid (CSF) partial volume fractions. In each scan session, the anatomical volume was aligned to the functional volume using AFNI. Each functional volume was spatially smoothed using a Gaussian filter with 3 mm FWHM.

The images from the first 5 timepoints (10 s) of the BOLD data were discarded to allow magnetization to reach a steady state. A binary brain mask was created using the skull-stripped anatomical data. For each slice, the mask was eroded by two voxels along the border to eliminate voxels at the edge of the brain [19]. For each run, nuisance terms were removed from the resting-state BOLD time series through multiple linear regression, with the following nuisance regressors [15]: i) mean, linear and quadratic trends, ii) six motion parameters estimated during image co-registration and their first derivatives, and iii) the mean BOLD signal calculated from WM and CSF regions and their first derivatives, where these regions were defined using partial volume thresholds of 0.99 for each tissue type and morphological erosion of two voxels in each direction to minimize partial voluming with gray matter. It is important to note that after the regression, the mean was added back to the BOLD time series. In processing resting-state data, it is a common practice to apply low pass filtering (typically with a 0.08 Hz cut-off frequency) [11]. However, as recent studies suggest that high frequency components in the BOLD signal contain useful information [30], we did not apply low pass filtering to the data for our default processing. We assessed the amount of head motion of each subject by first calculating the framewise displacement (FD) as defined by Power et al. [31] using the 6 motion parameter time courses. The overall amount of head motion was then obtained by averaging the FD across time and the values are listed in Table 1. There was not a significant correlation (r = 0.09; p = 0.80) between the motion metrics and the performance scores.

For each voxel, a percent change time series was then calculated [15], [19], [32], [33]. The mean value was first subtracted from the time series. Next, the resulting difference was divided by the mean value. The percentage change time series from the two resting-state runs were concatenated. We then converted the whole brain BOLD data for each subject to Talairach space. In the coarse registration step, a 12-parameter affine transformation matrix was estimated by registering the anatomical volume to the T1 template (TT_avg152T1+tlrc) using *3dAllineate* in AFNI. In the refinement step, a non-linear warping transformation was calculated using *3dQWarp*. The linear matrix and the non-linear warping transformation were then sequentially applied to warp the BOLD data into Talairach space, resulting in standardized data with 2 mm isotropic resolution.

We then computed the BOLD signal variability for each voxel, defined as the standard deviation of the percent change time series. For the assessment of connectivity, we adopted the anatomical parcellation in AFNI “TT_desai_dk_mpm+tlrc”. We selected ROIs within the parcellation for which the BOLD signal variability was found to be significantly correlated with the performance score (Table 2). Within each of these ROIs, the BOLD time courses were averaged. The averaged BOLD time courses were then correlated with every voxel within the brain. The relation between the fMRI metrics (BOLD signal variability and correlation) and the performance scores across subjects was assessed using linear regression.

**Table 2:** Regions of significant correlation (p<0.05, corrected for multiple comparisons using AlphaSim in AFNI, minimum cluster size  = 258 voxels) between the BOLD signal variability and performance scores across subjects.

Brain regions	Side	# of voxels	Peak coordinates (in LPS orientation)			Peak correlation with the performance score	
			x	y	z	r	p
**Basal ganglia**							
Caudate	L	11	6	−8	2	0.75	0.01
	R	34	−8	−12	4	0.87	0.001
Putamen	L	213	24	14	−4	0.89	6e-4
	R	176	−26	−10	−2	0.85	0.002
Pallidum	L	90	22	12	−2	0.83	0.003
	R	86	−18	2	−4	0.88	8e-4
Nucleus accumbens	L	29	6	−8	−4	0.9	4e-4
	R	37	−8	−6	−10	0.87	8e-4
**Other subcortical areas**							
Anterior hippocampus	L	80	22	6	−18	0.93	1e-4
Amygdala	L	159	16	−2	−12	0.94	5e-5
	R	28	−26	−2	−14	0.84	0.002
Thalamus	L	62	4	4	2	0.86	0.001
	R	149	−12	12	14	0.89	6e-4
**Frontal lobe**							
Superior frontal	R	334	−8	−24	54	0.97	3e-6
Lateral orbito frontal	L	154	18	−6	−16	0.95	3e-5
	R	88	−30	−24	−16	0.90	4e-4
Inferior frontal (pars opercularis)	R	32	−48	−8-	0	0.85	0.002
**Temporal lobe**							
Superior temporal	L	38	44	−4	−12	0.87	0.001
	R	160	−50	−8	0	0.86	0.001
Middle temporal	R	38	−54	0	−22	0.80	0.005
**Insula cortex**	L	160	34	−14	−8	0.87	0.001
	R	74	−28	−8	−12	0.81	0.005

Within each region, the peak correlation (and the associated p-value) with the performance score is provided for the purpose of qualitative assessment.

## Results

**Figure 2:**
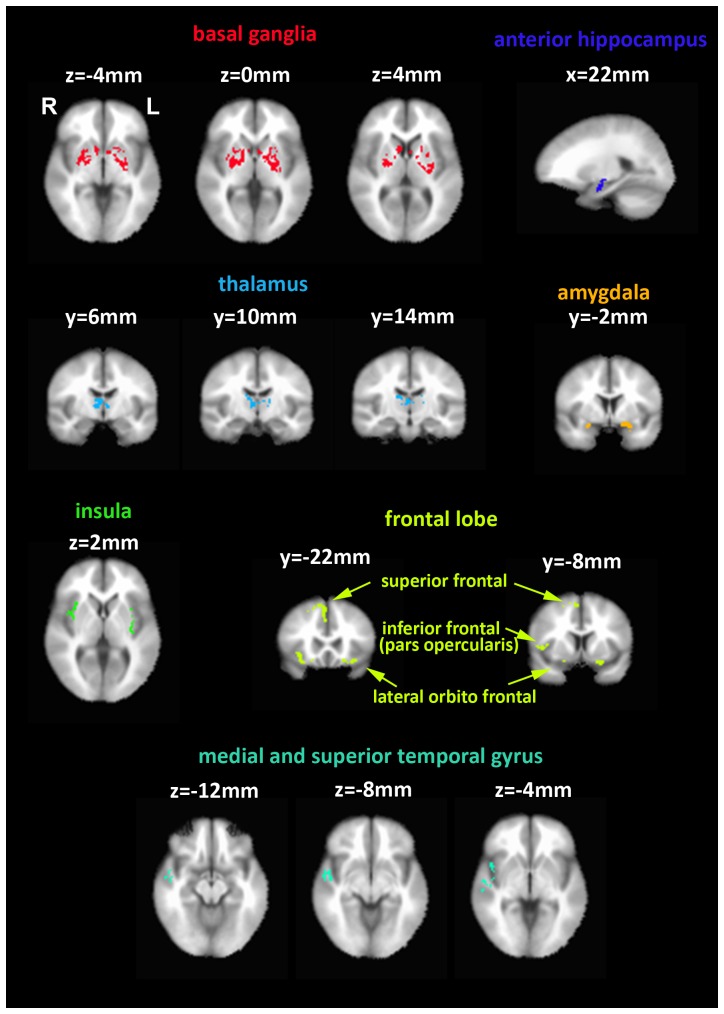
Whole brain map highlighting regions of significant correlation (p<0.05, corrected for multiple comparisons using AlphaSim in AFNI, minimum cluster size  = 258 voxels) between BOLD signal variability and performance scores across subjects.

Fig. 2 displays brain maps showing clusters that exhibited significant correlation between the voxel-wise BOLD signal variability and the performance scores across subjects. Significant correlations (p<0.05, corrected for multiple comparisons using a family-wise approach called AlphaSim [34], [35] in AFNI, minimum cluster size  = 258 voxels) were observed within the basal ganglia, left anterior hippocampus, amygdala, thalamus, right superior frontal gyrus, lateral orbito frontal cortex, pars opercularis of the right inferior frontal gyrus, right middle temporal gyrus, superior temporal gyrus, and insula cortex. In each of these regions, the BOLD signal variability was higher for the better performers. Table 2 lists the brain regions associated with each cluster. The whole brain map in Fig. S1 shows the correlation values between the BOLD signal variability and performance scores across subjects. To provide a qualitative view of the relation between BOLD signal variability and performance scores across subjects, we averaged the BOLD time courses within each cluster, and then calculated the BOLD signal variability of this average signal. Fig. S2 plots the BOLD signal variability from each cluster versus the performance score.

**Figure 3:**
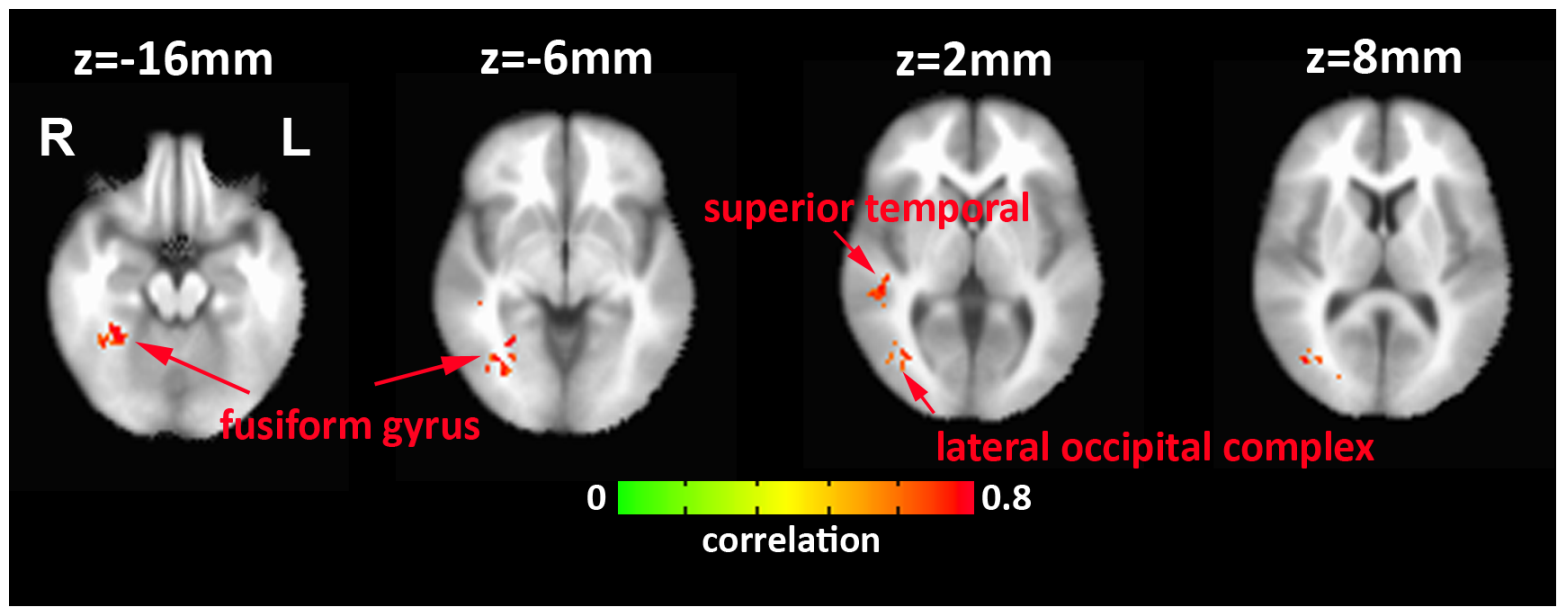
Whole brain correlation map showing regions that exhibit a significant correlation (p<0.05, corrected for multiple comparisons using AlphaSim in AFNI, minimum cluster size  = 258 voxels) between performance scores and functional connectivity with the left caudate.

To examine the relation between brain functional connectivity and performance scores, we used the ROIs listed in Table 2 and then extracted the associated anatomical ROIs from the AFNI “TT_desai_dk_mpm+tlrc” template [36] as seed regions (23 ROIs, mean size  = 971 voxels, range  = 93 to 4088 voxels) and computed the correlation between the average signal in each seed region and all other voxels in the brain. We converted the correlation values to z-scores using the Fisher z-transformation [37] and then correlated the z-scores with the performance scores. Fig. 3 displays whole brain maps showing regions for which the functional connectivity with the left caudate was significantly correlated with the performance scores across subjects. Significant relations (p<0.05, corrected for multiple comparisons using AlphaSim, minimum cluster size  = 258 voxels) were observed for the fusiform gyrus, lateral occipital complex and superior temporal sulcus regions in the right hemisphere. In each of these regions, the BOLD functional connectivity with the seed ROI increased with performance score. We did not observe significant relations using the other seed ROIs listed in Table 1.

## Discussion

We have shown that resting-state BOLD signal variability in multiple brain regions (basal ganglia, hippocampus, amygdala, thalamus, insula, and regions in the frontal and temporal lobes) is correlated with unsupervised spatial learning performance in an immersive VR environment. In addition, we found that the resting-state functional connectivity between the left caudate and right hemisphere areas associated with object recognition and visual perception is correlated with learning performance (Fig. 3).

In our experimental paradigm, subjects were not aware of the memory component of the task during the free exploration on day 1. Since subjects were learning location-object associations in an unsupervised fashion (there was no explicit instruction or reinforcement involved), we were able to highlight the unsupervised learning aspect of the task. In addition, this type of experiment has been used in the rodent literature [38]–[41] to examine unsupervised learning. Thus, the results of this study contribute to our understanding of the brain regions involved in unsupervised spatial learning.

Although the findings are correlational, the observed relation between BOLD signal variability and performance across multiple regions is consistent with the involvement of multiple aspects of behavior in the experimental paradigm, which required subjects to engage in exploration, unsupervised learning, memory, and decision-making. With regards to exploration, prior studies have demonstrated that basal ganglia circuits play a critical role in facilitating exploratory behaviors [42]–[46]. The thalamus is tightly coupled to the basal ganglia [47], [48], and the correlation between BOLD signal variability and performance in this region may reflect this close relationship.

The association between performance and BOLD signal variability in the anterior hippocampus, amygdala and temporal lobe reflects the role of these brain regions in various aspects of memory and learning [49]–[56]. For example, activity in the anterior hippocampus has been shown to be related to associative memory [57], [58], while activity in the amygdala has been linked with associative and emotional learning [49], [53]. Together with the prefrontal cortex and hippocampus, the amygdala contributes in generating motivational signals to the ventral striatum for enhancing learning and incorporating episodic information [56]. Furthermore, the middle and superior temporal gyri in the right hemisphere are thought to belong to a neural network that supports spatial learning [54], [55]. The other regions identified in our study (insula, right superior frontal, lateral orbito-frontal and pars opercularis of the right inferior frontal gyri) have been shown to be associated with processes of self-representation and decision making [51], [59]–[64]. In summary, brain regions associated with spatial and episodic memory appears to be involved during unsupervised learning.

We found that learning performance was associated with functional connectivity between the left caudate and brain regions (lateral occipital complex, fusiform gyrus, and superior temporal sulcus) responsible for visuospatial object processing and attention [65]–[71]. Given the basal ganglia's role in exploration, these findings suggest that tighter integration of the brain systems responsible for exploration and visuospatial processing may be critical for learning in a complex environment.

In resting-state fMRI, one of the major networks that has been identified is the Default Mode Network (DMN) [11], [72]. Brain activity in the DMN was found to decrease during task performance and is thought to be a network that mediates the resting-state [73]. Hampson et al. [3] found that the connectivity in the DMN is associated with working memory performance. However, in the current study, we did not identify an association between the DMN and unsupervised learning performance. Further investigations examining the relationship between working memory and unsupervised learning would therefore be useful.

In the present study, we found that the correlation of connectivity associated with object recognition regions was observed only for the left basal ganglia, but not the right. In comparing our results with those of Vo et al. [1], we note that the findings of the prior study suggest a link between performance and structural connectivity (i.e. white matter tracts), while our current findings show that this link is also observed for functional connectivity measures based on intrinsic dynamic fluctuations. Both studies enrolled only right-handed subjects and observed that the link was more pronounced for structures within the left basal ganglia. The basis for this lateralization effect across studies needs to be further explored.

A potential limitation of the current study is that the findings were correlational, a property shared with a number of other recent studies that have examined the relation between intrinsic fMRI activity and behavioral performance [2], [3], [10]–[12], [74]. In general, these types of studies can be considered to lay the foundation for further studies that can more clearly elucidate the link between resting-state activity and behavior. For example, the ROIs identified in the current study can be used to guide the design of future studies aimed at deepening our understanding of the role of the basal ganglia in unsupervised learning.

In the current work, we were able to scan 10 subjects from a previously published study [23] that had a relatively small sample size (n = 13). The sample size used is similar to those found in three prior studies relating resting state activity to behavior [3], [12], [75], which used sample sizes of 9 and 14. It is possible that the sample size may have limited the ability of this study to detect brain regions in which the resting-state brain activity exhibits a weaker relation to unsupervised learning performance. Thus, this study can be considered to have identified the brain regions with the strongest correlation to unsupervised learning performance, with the distinct possibility that future studies will identify secondary regions that have a weaker correlation.

Recently, an increasing number of studies have examined the self-similarity of brain activity at multiple temporal scales [75]–[85]. Such scale-free or fractal time dynamics are typically long memory processes exhibiting a 1/f frequency spectrum, and have been found to be related to disease and cognitive performance [75], [86], [87]. In particular, Wink et al. have shown that response time in a fame decision/facial encoding task was inversely correlated with the mean Hurst exponent in the inferior frontal cortex calculated using resting-state fMRI data acquired after the task [75]. Further studies to investigate the relationship between the unsupervised learning performance and monofractal (e.g. Hurst exponent) or multifractal (e.g. Hölder exponent) dynamics of resting-state fMRI signals are warranted.

In this study, we considered measures of BOLD signal variability and connectivity over the course of two eight-minute resting-state runs. Recent studies have shown that significant variations in functional connectivity can occur over the length of a typical resting-state run [88]. Further studies to examine how dynamic variations in functional connectivity are related to unsupervised learning would be useful.

The VR experiment and the MRI scan dates in our study were spaced about 6 to 12 months apart. The fact that we were able to observe significant correlations between the performance scores and fMRI measures with a substantial temporal spacing between measures suggests that unsupervised learning ability and the associated resting-state brain activity may both be relatively stable traits. Resting-state fMRI measures may therefore prove to be a useful method for identifying individuals who are likely to perform better in unsupervised learning environments.

## Supporting Information

Figure S1 Whole brain map showing correlation values between the BOLD signal variability and performance scores across subjects (p<0.05, corrected for multiple comparisons using AlphaSim in AFNI, minimum cluster size  = 258 voxels).
(TIF)

Figure S2 BOLD signal variability (calculated using the averaged BOLD signal within each significant cluster) versus performance score plotted for the significant clusters identified in Table 2.
(TIF)
