# Supplementary figures and images for: Resting-State fMRI Activity Predicts Unsupervised Learning and Memory in an Immersive Virtual Reality Environment
Source: PLoS One. 2014 Oct 6;9(10):e109622. doi: 10.1371/journal.pone.0109622 (PMC4186845; doi:10.1371/journal.pone.0109622)

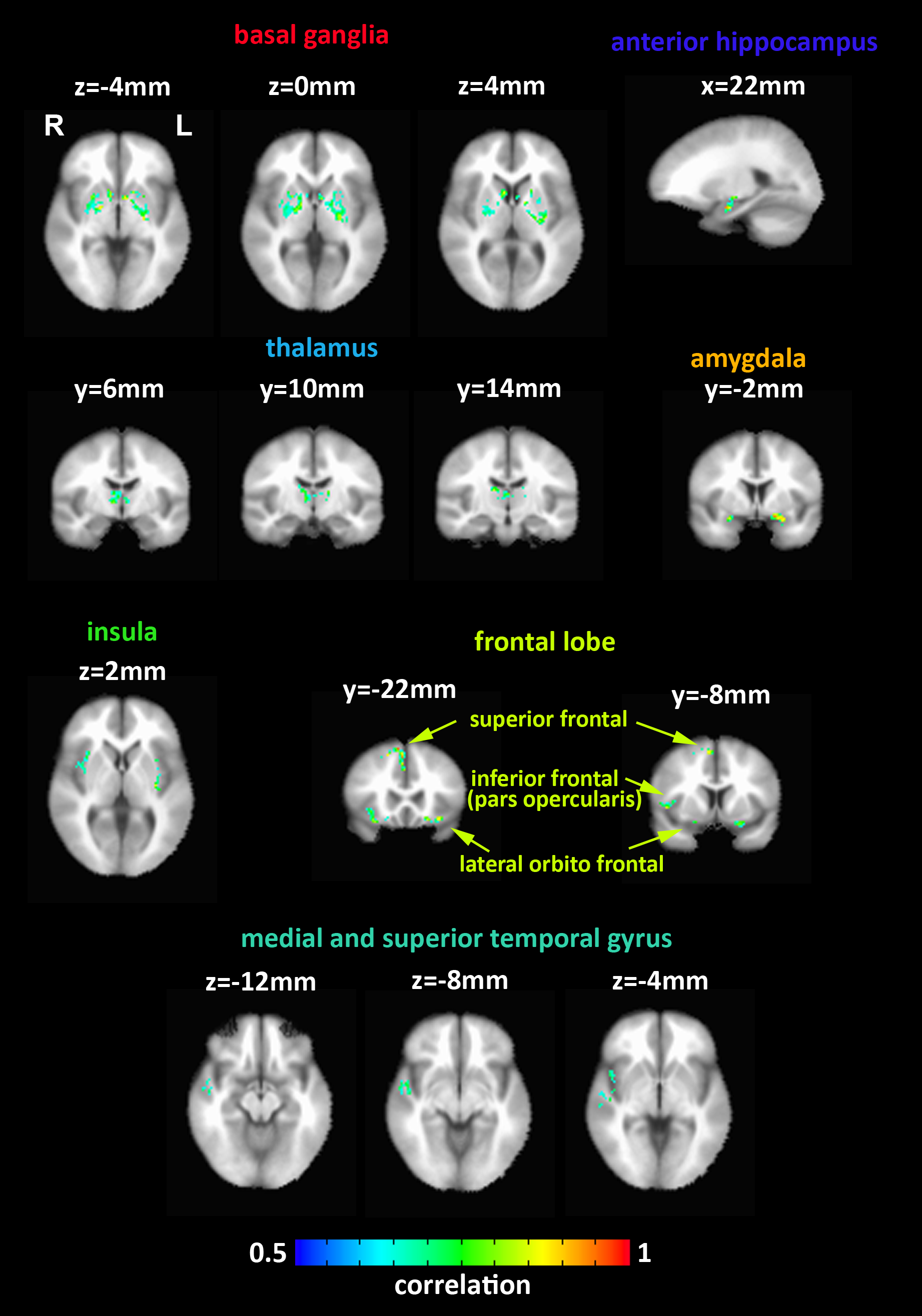

Supplement: Figure S1 — Whole brain map showing correlation values between the BOLD signal variability and performance scores across subjects (p<0.05, corrected for multiple comparisons using AlphaSim in AFNI, minimum cluster size = 258 voxels). (TIF) [file pone.0109622.s001.tif]

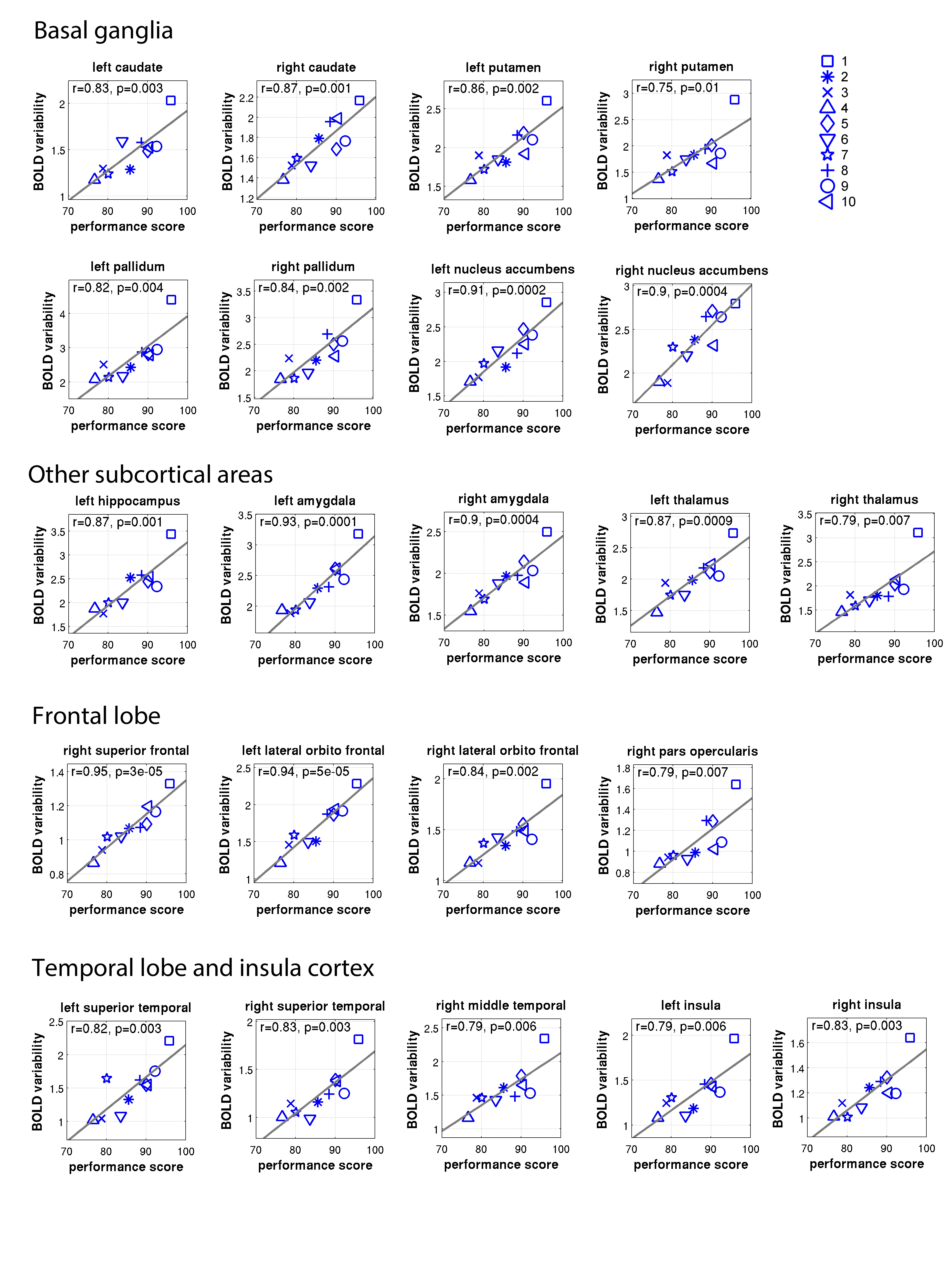

Supplement: Figure S2 — BOLD signal variability (calculated using the averaged BOLD signal within each significant cluster) versus performance score plotted for the significant clusters identified in Table 2. (TIF) [file pone.0109622.s002.tif]
